# Bilateral Hypoglossal Nerve Palsy due to Brainstem Infarction: A Rare Presentation of Presumed Pyogenic Meningitis

**DOI:** 10.1155/2018/8270903

**Published:** 2018-09-16

**Authors:** A. G. T. A. Kariyawasam, C. L. Fonseka, S. D. A. L. Singhapura, J. S. Hewavithana, H. M. M. Herath, K. D. Pathirana

**Affiliations:** ^1^Registrar in Medicine, University Medical Unit, Teaching Hospital Karapitiya, Sri Lanka; ^2^Consultant Physician, Department of Internal Medicine, Faculty of Medicine, University of Ruhuna, Sri Lanka; ^3^Consultant Physician, University Medical Unit, Teaching Hospital, Karapitiya, Galle, Sri Lanka; ^4^Professor in Neurology, Department of Internal Medicine, Faculty of Medicine, University of Ruhuna, Sri Lanka

## Abstract

**Background:**

Cranial nerve palsies are well-known complications of basal meningitis, especially in patients with tuberculous meningitis. However, a minority of bacterial meningitis gets complicated with cranial nerve palsies. Although cerebral infarctions are known to occur with acute bacterial meningitis, infarctions occurring in the brainstem are infrequently described.

**Case Presentation:**

We report a 46-year-old healthy female who presented with dysarthria with fever, headache, and vomiting and was diagnosed to have acute pyogenic meningitis complicated with a brainstem infarction resulting in bilateral hypoglossal palsy. Her MRI revealed an infarction in the lower part of the medulla oblongata, probably involving the bilateral hypoglossal nuclei.

**Conclusion:**

Isolated bilateral hypoglossal nerve palsy is an extremely rare cranial nerve palsy, secondary to pyogenic meningitis. To our knowledge, this should be the first reported case of isolated bilateral hypoglossal nerve palsy due to a brainstem infarct in the background of pyogenic meningitis.

## 1. Introduction

Despite the current advanced treatment, meningitis still carries a substantial morbidity and mortality [1]. Fever, headache, and neck stiffness are considered as the hallmarks of meningitis. Presence of focal signs in meningitis points towards an unfavourable outcome [2]. Cranial nerve palsies are one of such focal signs associated with meningitis. In general, the cranial nerve involvement in meningitis is associated with a higher risk of mortality and disability and 5-10% of patients with acute bacterial meningitis can get complicated with cranial nerve involvement in the early course of the illness [3]. Multiple cranial nerve involvement is usually considered as an indicator of basilar meningitis and is thought to be a result of compression caused by oedematous brain or meningeal inflammation resulting in perineuritis. Third, fourth, sixth, and seventh nerves are the commonest nerves to get affected [3] and the involvement of the hypoglossal nerve is very rarely described in literature.

We herewith report a rare case of bilateral hypoglossal nerve palsy due to posteromedial infarction in the medulla oblongata secondary to acute bacterial meningitis. As far as we are aware, this is the first reported case of isolated bilateral hypoglossal nerve palsy, secondary to acute bacterial meningitis.

## 2. Case Presentation

**Figure 1:**
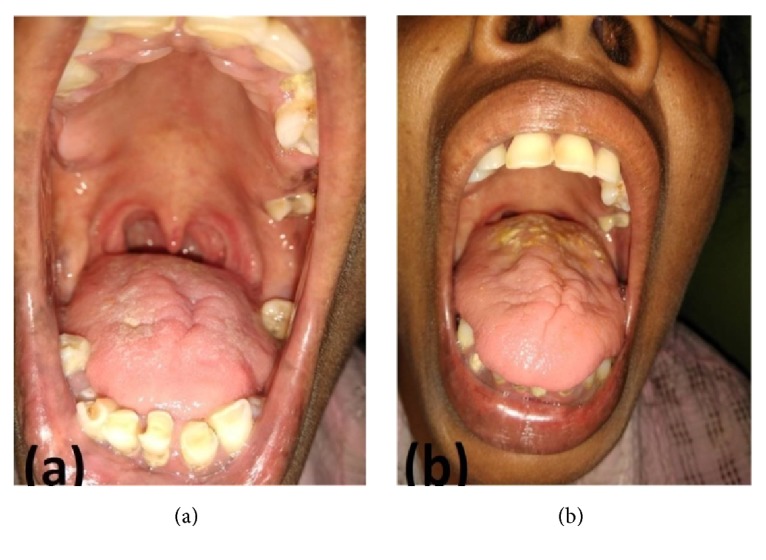
Bilateral tongue wasting: (a) resting position; (b) on protrusion.

A 46-year-old previously healthy female developed an insidious onset severe persistent headache, most prominent in the occipital region lasting for 10 days. Six days after the onset, she experienced dysarthria and a difficulty in moving her tongue within the mouth with a difficulty in eating and drinking. She did not complain of nasal regurgitation of food or nasal quality of speech. After admission, she was found to have a high-grade fever. She was otherwise healthy and denied symptoms of cough, decreased appetite, weight loss, or past history of tuberculosis. On admission, she was found to be ill with elicitable neck stiffness. Neurological examination revealed bilateral hypoglossal nerve palsy with marked tongue atrophy, more prominent in the left side (Figure 1) with tongue fasciculations and without other cranial nerve palsies or pyramidal weakness. Her eye movements were saccadic with a broad-based ataxic gait without other signs of cerebellar involvement.

Her blood tests revealed a haemoglobin of 12.5g/dl with a neutrophil leukocytosis (19,000/*µ*L; 92.2% of neutrophils) with elevated ESR (100 1st Hr) and CRP (195 u/L). Her blood cultures were negative. Noncontrast CT brain did not reveal any abnormality. Cerebrospinal fluid (CSF) biochemistry revealed significant elevation of protein (111 mg/dL) with 59 polymorphs and 8 lymphocytes per cubic millimetre with reduced CSF glucose (29 mg/dL). CSF for GeneXpert for tuberculosis and staining for acid-fast bacillus (AFB) and fungal and atypical cells were negative. Pyogenic, mycobacterial, and fungal CSF cultures were negative and CSF for Meningococcus, Haemophilus, and Pneumococcus antigens were also negative. Her chest radiograph did not reveal any changes suggestive of pulmonary tuberculosis or sarcoidosis. Syphilis (VDRL & THPA), HIV serology, and autoimmune markers for vasculitis (rheumatoid factor, ANA (IF), and p & c-ANCA) were negative.

**Figure 2:**
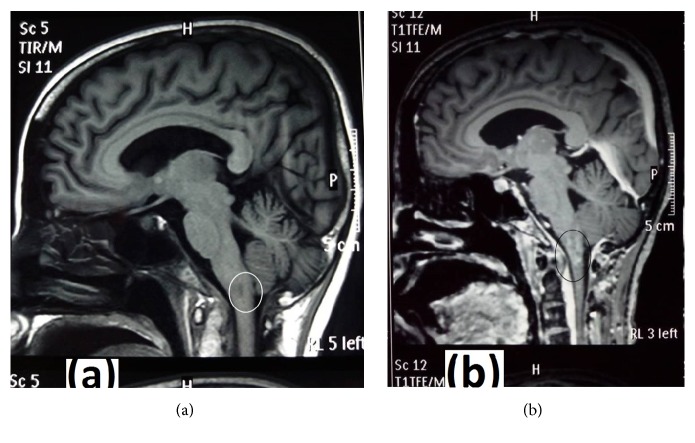
T1-weighted MRI of brainstem involving medulla oblongata demonstrating an infection: (a) without contrast; (b) postcontrast.

We initiated her on empirical treatment as for pyogenic meningitis with ceftriaxone and vancomycin for which she had a gradual improvement of general status with improvement of fever, meningism, gaze, and gait abnormalities while tongue weakness and atrophy persisted. Since we considered tuberculous meningitis as a possibility, we deferred treatment with steroids. Her rapid recovery in the absence of steroids or antituberculous drugs further supported our presumed diagnosis of pyogenic meningitis. Subsequently, she underwent MRI of brain and brainstem, which revealed a posteromedial infarction in the lower part of the medulla oblongata without leptomeningeal enhancement and did not show a significant cerebral oedema (Figure 2). At the end of three weeks of antibiotics, inflammatory markers and repeat CSF analysis reached normal levels. After discharge, we reviewed her at one month and three & six months and she was free of fever with good general condition and had normal inflammatory markers. However, she had persistent tongue atrophy with difficult speech from which she was gradually recovering with the help of physiotherapy.

## 3. Discussion

Hypoglossal nerve palsy (HNP) is an uncommon cranial nerve palsy comparing to other commoner cranial nerves such as 3rd, 6th, and 7th cranial nerves [4]. Kean et al. in a case series described 100 patients with HNP either isolated or together with other cranial nerve palsies. In nearly 50 percent, HNP was secondary to either primary or secondary intracranial malignancy [5]. Therefore, it was concluded that HNP is a sign of intracranial malignancy indicating poor prognosis. Some years later, Panagariya et al. described a case series containing 12 clinical cases of isolated HNP and found that 33% had intracranial tuberculosis which were treated with antituberculous therapy. Other causes were sarcoidosis (8%), idiopathic (25%), and atlantooccipital dislocation (8%) and the overall recovery rate was nearly 60% [6].

Rocholt et al. described a patient with meningitis due to* Neisseria Meningitidis* complicated with obstructive hydrocephalus who had isolated unilateral HNP which resolved over 5 months [3]. Although unilateral HNP was infrequently reported with pyogenic meningitis, we could not find cases where bilateral HNP was reported.

A recent retrospective study was conducted in patients affected with acute bacterial meningitis (ABM) or tuberculous meningitis (TBM); the predictive value of each neurological sign on the diagnosis of above was analysed. They observed that the presence of cranial nerve palsies was the most important neurological predictor favouring TBM over ABM (OR = 1.980, CI 95%: 1.161-3.376) [2]. Since tuberculosis is commonly encountered in Sri Lanka, we strongly suspected TB meningitis. But, considering her acute presentation, absence of constitutional symptoms, and CSF finding of polymorphic cell predominance, we initiated treatment as pyogenic meningitis. We continued to investigate further to exclude possible tuberculosis. The GeneXpert test in CSF which is nearly 60% sensitive and CSF AFB (sensitivity 10-20%) and TB cultures (sensitivity 50-60%) [7] were all negative. Our patient did not show meningeal enhancement or basal exudates in MRI brain, which is the most sensitive radiological feature, found in 90% of CNS tuberculosis [8].

With treatment, our patient showed a significant clinical improvement and inflammatory markers became normal. In addition, repeat CSF analysis two weeks later showed complete resolution of CSF abnormalities. All these findings stabilized the diagnosis of acute pyogenic meningitis. We attributed the uncommon presentation of bilateral tongue involvement to a posteromedial medullary infarction which was observed in the MRI scan of the brain (Figure 2).

For several decades, attention has been paid to the neurological complications of meningitis including cerebral infarctions. Diederik van de Beek et al. in a review described that 15-20% of community-acquired bacterial meningitis is complicated with infarctions of the brain matter [9].

According to another Danish population-based cohort study, 14% infarctions were observed in the course of ABM [10]. Many similar studies have proven that cerebral infarctions do occur as a complication in a significant proportion of patients with ABM [11]. Instances where such ischemic strokes occurring in the brainstem though rare are reported in the literature [12–14]. The patient had significant atrophy of the tongue. We assumed that this is due to denervation atrophy secondary to the brainstem infarction, although it is an uncommon finding in acute cases, due to the fact that the subacute nature of presentation atrophy could be expected. The hypoglossal nerve is the sole motor innervator of the tongue; denervation atrophy of the tongue can result in significant changes of the tongue [15].

Apart from the involvement of bilateral HNP, our patient had other brainstem signs such as saccadic eye movements and cerebellar ataxia which got completely resolved with treatment. According to an animal experimental study carried out to explain the pathophysiology of eye saccades, it was suggested that the saccade palsy could have resulted from an insult to the lower pons [16]. This is supposed to be due to damage to caudal paramedian-pontine reticular formations (PPRF). When PPRF is damaged bilaterally, inputs from caudal PPRF to the rostral interstitial nucleus of the medial longitudinal fasciculus are greatly affected through the direct ascending anatomic pathway between them. This is supposed to result in oculomotor signs [17]. Although MRI did not show a definite pontine lesion in our patient, it could occur due to brain oedema or incomplete ischemia extending to the lower pons [17]. Complete recovery of other brainstem signs suggests the possible transient damage of other brainstem areas due to oedema or incomplete ischemia as a result of inflammatory response induced by the basilar meningitis.

## 4. Conclusions

Isolated bilateral hypoglossal nerve palsy is extremely rare cranial nerve palsy secondary to pyogenic meningitis. Brainstem infarcts involving the medial medullary region of the medulla oblongata can lead to a presentation of bilateral hypoglossal nerve palsy. To our knowledge, this is the first reported case of bilateral hypoglossal nerve palsy due to a brainstem infarct in the background of pyogenic meningitis.

## Data Availability

All relevant data are included in the manuscript.
